# Molecular Basis of Crops and Fruit Plants in Response to Stress

**DOI:** 10.3390/plants12223813

**Published:** 2023-11-09

**Authors:** Jose Helio Costa, Rafael de Souza Miranda

**Affiliations:** 1 Functional Genomics and Bioinformatics, Department of Biochemistry and Molecular Biology, Federal University of Ceara, Fortaleza 60451-970, Ceara, Brazil; 2 Non-Institutional Competence Focus (NICFocus) ‘Functional Cell Reprogramming and Organism Plasticity’ (FunCROP) (Coordinated from Foros de Vale de Figueira), 7050-704 Alentejo, Portugal; 3 Plant Science Department, Federal University of Piauí, Teresina 64049-550, Piauí, Brazil; 4 Postgraduate Program in Agricultural Sciences, Campus Professora Cinobelina Elvas, Federal University of Piauí, Bom Jesus 64900-000, Piauí, Brazil

**Keywords:** cultivars, genotypes, resistance, tolerance, endophytes

## Abstract

This editorial summarizes the main scientific contributions from 11 papers comprising the Special Issue (SI) “Molecular Basis of Crops and Fruit Plants in Response to Stress”. Here, we collected papers from different research groups encompassing molecular studies from monocots (ginger, rice, maize) and eudicots (common hazel, cowpea, pepper, soybean, tomato) species submitted to abiotic stresses as heat, cold, salt, drought, and heavy metals or biotic stresses induced by different viruses, such as *BPEV*, *PepGMV*, *PMMoV*, and *TEV*. These studies explored different aspects of molecular mechanisms involved in plant stress tolerance, establishing comparative analyses among genotypes/cultivars to identify potential molecular markers of stresses that are now available for future application in biotechnological studies. This SI presents a collection of advanced concepts and emerging strategies for readers and researchers aiming to accelerate plant breeding.

## 1. Introduction

The Special Issue (SI) “Molecular Basis of Crops and Fruit Plants in Response to Stress” was planned to advance into different aspects of DNA, RNA, proteins, and metabolites associated with biochemical and physiological responses to stress resistance/tolerance. Recently, the continuous progress in genomics, transcriptomics, proteomics, and metabolomics boosted the identification of genes, proteins, and metabolites applied in breeding programs. However, because of the large diversity among plant species as well as genotypes/cultivars in each species, which may differ in adaptation and acclimatization to environmental changes or recalcitrant soils, plant breeders continue searching for alternative strategies to obtain plants resistant to variable stress conditions conducting to higher efficiency and productivity. Therefore, this SI focused on the invitation of researchers to present new findings regarding genes, proteins, metabolites, endophytic/plant interaction, and mechanisms of cultivars/genotypes involved in stress tolerance. Different papers covering some monocot and eudicot species under abiotic (cold, salt, drought, and heavy metals) or biotic (*BPEV*, *PepGMV*, *PMMoV*, and *TEV*) stresses were collected.

## 2. Overview of SI

The articles published in this SI covered the identification and characterization of transcription factors associated with stress resistance [1,2,3], beneficial endophytic/plant interaction [4,5], and molecular mechanisms involved in crops and fruit plants to stress tolerance [6,7,8,9,10,11].

### 2.1. Exploring the Function of Transcription Factors in Plant Stress Resistance

Transcription factors are recognized as proteins that interact specifically with DNA sequences regulating the expression of a single gene or a set of genes involved in plant development or stress responses. In this SI, Su et al. [1] analyzed the gene family of mitochondrial transcription termination factor (mTERF) in tomatoes, identifying 28 mTERF gene members through a genome-wide mining analysis. All gene members contained responsive elements associated with hormone or environmental stresses (drought, salt, and cold), which corroborated the gene expression analyses. In addition, the gene-silencing approach revealed a key role of mTERF13 gene members in abiotic stress resistance [1].

In a second paper, Filyushin et al. [2] identified a new A2-type dehydration-responsive element-binding (DREB) transcription factor in the maize genome. This gene, named ZmDREB2.9, showed two alternative splicing events generating short (ZmDREB2.9-S) and long (ZmDREB2.9-L)-protein isoforms, differentially expressed in different plant organs. Expression analyses of ZmDREB2.9 and A2-type ZmDREB2.1–2.8 genes under abiotic stresses (drought and cold) and abscisic acid treatment provided new insights into gene function and suggested their high potentiality for breeding programs in stress tolerance [2].

Finally, in a third study, Jiang et al. [3] explored the evolution and expression profiling of ginger’s heat shock transcription Factor (HSF) gene family during developmental and abiotic stress responses. The authors identified 25 HSF gene members in the ginger genome, which were divided into three groups (HSFA, HSFB, and HSFC) compared to Arabidopsis HSF genes. However, a more detailed investigation showed the highest ginger HSF gene collinearity with HSF genes from several monocots than Arabidopsis, a dicot model. Furthermore, ginger HSF genes revealed differential expression regarding different tissues with the majority of gene members showing heat and drought stress responses [3].

### 2.2. Endophytic Interaction Promoting Plant Stress Resistance

Endophytes are bacterium or fungi living in plant organs or cells in a mutualistic relationship. Although some endophytes are recognized to improve host development or a plant’s capacity for stress resistance, molecular insights into endophyte/plant interactions need to be clarified. In this SI, two studies explored the role of endophyte interactions in Solanaceae plants, such as pepper [4] and tomato [5].

In pepper, Samaniego-Gámez et al. [4] analyzed the ability of *Bacillus* strains to promote ISR (Induced systemic resistance) in plants infected with pepper golden mosaic virus (PepGMV). The authors observed that pepper inoculation with *Bacillus* subtilis K47, *Bacillus* cereus K46, and *Bacillus* sp. M9 reduced the PepGMV infection. Curiously, this response was associated with the upregulation of plant defense genes such as CcNPR1, CcPR10, and CcCOI1, corroborating the ISR promoted by these *Bacillus* strains.

In tomatoes, Badawy et al. [5] evaluated the capacity of endophytic bacteria (Micrococcus luteus and Enterobacter cloacae) to alleviate heavy metal (Cd and Ni) stress. Interestingly, the researchers reported a strong resistance of tomato plants to Cd and Ni stresses mediated by endophytic bacteria. This response involved the activation of enzymatic and non-enzymatic antioxidant defenses and osmoregulation by increasing proline, mineral content, and related regulatory genes.

Therefore, these papers evidenced that endophytes can improve the resistance of Solanaceae plants to both biotic [4] and abiotic stress [5].

### 2.3. Different Approaches Covering Molecular Mechanisms Involved in Stress Tolerance

Miranda et al. [6] explored the growth and biochemical parameters in leguminous plants such as soybeans and cowpeas to select cultivars with superior performance under drought during the vegetative stage. The authors observed that, in general, soybean cultivars proved to be more resistant to drought than cowpea cultivars. Among seven cultivars evaluated in each species, BÔNUS8579IPRO and TMG1180RR from soybean and Xique-xique from cowpea revealed the highest performance under drought. However, the mechanisms involved in stress tolerance were different between species, with soybean cultivars showing increased amino acids and proline contents while cowpea cultivars had elevated photosynthetic pigments and maintenance of water content.

In a second approach, Aziz et al. [7] investigated early cell reprogramming in rice using a salt-tolerant contrasting model of two genotypes: Pokkali tolerant and IR29 susceptible. For these analyses, the authors studied a specific gene set associated with the control of ROS formation (AOX, UCP, and PTOX), impact on ATP production (PFK, ADH, and COX), and antioxidant enzymes over a period of 24 h. Interestingly, the tolerance of the Pokkali genotype was linked to the higher mRNA levels of AOX, PFK, and ADH, indicating higher glycolysis activity (PFK) and fermentation (ADH) to obtain rapid energy as well as ROS balancing by AOX. In addition, this genotype revealed specific gene responses of the AsA-GSH cycle suggesting that the salt tolerance mechanism in rice involves the antioxidant ascorbate.

In another study, Solar et al. [8] evaluated the variability of the kernels’ phenolic contents in 5 hazelnut cultivars originating from European regions with different climatic conditions. After analyses, they identified thirteen phenols in the hazelnut kernels divided into seven flavanols, two hydroxybenzoic acids, three flavonols, and one dihydrochalcone. Remarkably, the region/climatic condition influenced the type and accumulation of phenolic compounds. The catechin and total flavanols were identified as having the highest content in Spain and northern Italy cultivars. However, flavanols and flavonols were detected mainly in cultivars from regions with high solar irradiation, while hydroxybenzoic acids prevailed in cultivars from mountain areas (altitude). In addition, cultivars from regions with reduced annual rainfall (indicating hydric stress) correlated negatively with dihydrochalcone, while soil pH correlated positively with phenolic compound. Overall, this paper suggests that controlling environmental conditions is helpful in generating the metabolite architecture in hazelnut kernels.

De Aguiar et al. [9] extensively explored transcriptome data of pepper plants to identify the contribution of ascorbate (Asc) biosynthesis pathways during fruit development, stresses, and phytohormone treatments. The authors reported that the L-galactose pathway was found to be the main Asc biosynthesis pathway in all studied conditions, while the myo-inositol and L-gulose pathways seemed to play a secondary role. The extensive transcriptome analyses of 21 genes from Asc biosynthesis involving variable genotypes/cultivars under biotic (*BPEV*, *PMMoV*, and *TEV*) and abiotic (heat, cold, salt, and mannitol) stresses and during fruit development revealed GGP2, GME1 and 2, and GalLDH members from L-galactose pathway as gene candidates to breeding programs, aiming to increase the ascorbate content in peppers and other crops.

Setubal et al. [10] studied the effect of nitrogen fertilization and water stress on plant growth, nutrient dynamics, and other parameters in several time points (16, 23, 30, 37, 44, 58, 65, 79, and 86 days after emergence) during soybean development. Enquiringly, the authors observed that the most drought-induced damages occurred at the reproductive stage (R1 to R6) impacting the seed yield. In addition, despite nitrogen fertilization of soybean plants improved N accumulation at maximum level, it did not contribute to substantial seed yield improvements under drought compared to control plants. Thus, these data indicated that the cost of nitrogen fertilization would be economically viable only in soybean plants under full irrigation.

In the last approach, Li et al. [11] reviewed the mechanisms involved in maize salt tolerance. The authors explored relevant plant defense mechanisms associated with salinity tolerance including the following topics: osmolyte accumulation and signaling pathways, antioxidant enzymes, reactive oxygen species, phytohormones, and the role of Na^+^, K^+^, and Cl^−^ in salt tolerance of maize plants. Also, the authors presented an interesting view to counteract salinity damage in crops.

## 3. Conclusions

In an integrated view, the studies with gene families of transcription factors such as mTERF in tomato [1], DREB in maize [2], and HSF in ginger [3] revealed the involvement of specific gene members in different stress conditions. Specific endophytes can improve the resistance of pepper [4] and tomato [5] plants to biotic or abiotic stresses. Miranda et al. [6] identified soybean and cowpea cultivars with superior performance under drought. Aziz et al. [7] revealed the critical involvement of glycolysis, fermentation, and alternative oxidase to salt stress tolerance in rice. Solar et al. [8] identified the influence of different climatic conditions on phenolic composition from European common hazel cultivars. De Aguiar et al. [9] identified specific gene members from the L-galactose pathway that could be used in molecular breeding programs to increase the ascorbate content. Setubal et al. [10] observed that nitrogen fertilization is inefficient for seed yield in soybeans under drought. Finally, Li et al. [11] revised different mechanisms associated with maize salt tolerance.

## Data Availability

All papers cited in this editorial are available in the following link: https://www.mdpi.com/journal/plants/special_issues/crop_fruit_stress (accessed on 8 October 2023).
